# Comparison of Bayesian Clustering and Edge Detection Methods for Inferring Boundaries in Landscape Genetics

**DOI:** 10.3390/ijms12020865

**Published:** 2011-01-25

**Authors:** Toni Safner, Mark P. Miller, Brad H. McRae, Marie-Josée Fortin, Stéphanie Manel

**Affiliations:** 1 Laboratory of Alpine Ecology, Equipe Population Genomics and Biodiversity, UMR CNRS 5553, BP 53, University Joseph Fourier, 38041 Grenoble Cedex 9, France; E-Mail: tsafner@gmail.com; 2 Department of Plant Breeding, Genetics and Biometrics, Faculty of Agriculture, University of Zagreb, Svetosimunska 25, 10000 Zagreb, Croatia; 3 Department of Biology, Utah State University, 5305 Old Main Hill, Logan, UT 84321, USA; E-Mail: markperrymiller@gmail.com; 4 The Nature Conservancy, 1917 1st Ave, Seattle, WA 98101, USA; E-Mail: mcrae@circuitscape.org; 5 Department of Ecology & Evolutionary Biology, University of Toronto, Ontario, M6R 2R8, Canada; E-Mail: fortinmj@gmail.com; 6 Laboratory of Population Environment Development, UMR 151 UP/IRD, University Aix-Marseille I, 3 place Victor Hugo, 13331 Marseille Cedex 03, France

**Keywords:** landscape genetics, genetic boundaries, spatial Bayesian clustering, edge detection methods

## Abstract

Recently, techniques available for identifying clusters of individuals or boundaries between clusters using genetic data from natural populations have expanded rapidly. Consequently, there is a need to evaluate these different techniques. We used spatially-explicit simulation models to compare three spatial Bayesian clustering programs and two edge detection methods. Spatially-structured populations were simulated where a continuous population was subdivided by barriers. We evaluated the ability of each method to correctly identify boundary locations while varying: (i) time after divergence, (ii) strength of isolation by distance, (iii) level of genetic diversity, and (iv) amount of gene flow across barriers. To further evaluate the methods’ effectiveness to detect genetic clusters in natural populations, we used previously published data on North American pumas and a European shrub. Our results show that with simulated and empirical data, the Bayesian spatial clustering algorithms outperformed direct edge detection methods. All methods incorrectly detected boundaries in the presence of strong patterns of isolation by distance. Based on this finding, we support the application of Bayesian spatial clustering algorithms for boundary detection in empirical datasets, with necessary tests for the influence of isolation by distance.

## 1. Introduction

In spatial ecology, a boundary is a region of abrupt change in a map of biological variables. Boundaries are of interest because their locations reflect underlying biological, physiological or social processes [1], including barriers to dispersal. Identification of genetic boundaries has recently received considerable attention in the fields of population genetics and phylogeography, especially in systems where species are either continuously or patchily distributed and where discrete populations are not easily defined. The detection of genetic boundaries may help to determine the underlying generating factors such as important historical events [2] or ongoing barriers to gene flow (e.g., [3–5]). Therefore, detecting genetic boundaries can help resolve population structure and improve understanding of population connectivity for conservation purposes [6], as well as allowing resource managers to better identify important habitats for preserving genetic variation within species (e.g., [7]).

Two general families of techniques are currently used to identify boundaries in population genetics [8]: Bayesian clustering algorithms and edge detection techniques. Bayesian clustering algorithms intrinsically strive to identify discrete sets of individuals based on the analysis of the multilocus genotypes [9–11]. They simultaneously delineate clusters of individuals based on the analysis of individual genotypes and assign individuals to the identified cluster where their posterior probability is highest. Although not specifically designed to do so, these analyses can all be interpreted in a spatial context, and as a by-product, individual clustering memberships can be used to identify potential genetic discontinuities across landscapes (e.g., [1,2]). Individuals can then be mapped with symbols reflecting cluster membership, and these symbols can be overlaid with maps of landscape features. Bayesian clustering methods have recently been enhanced to incorporate geographical locations of individuals in prior distributions [14–18]. In spatial models, the probability that two individuals belong to the same cluster is influenced by the geographic distance between them whereas geographic proximity is ignored in aspatial models.

Edge detection techniques directly identify areas where changes in variables occur from the analysis of allele frequency data [19]. Edge detection approaches such as Monmonier’s algorithm [20] and wombling [21,22] have a rich history of use in geography and ecology (see [23], and algorithms for applying them to genetic data have also been developed [24–27]). Some authors have detected genetic boundaries between populations using Monmonier’s algorithm [24,28,29], while Segelbacher *et al.* [30] used wombling at the individual level to detect genetic boundaries in capercaillie (*Tetrao urogallus*) populations.

Effective conservation and management strategies depend on our ability to correctly identify boundaries in genetic data. Although studies have compared the relative performance of Bayesian clustering methods ([31] for aspatial Bayesian clustering methods; [17,32] for spatial methods), none have explicitly compared the performance of clustering methods and edge detection methods using genetic data. Although Fortin and Drapeau [19] compared clustering and wombling-based methods using plant community data, such data respond strongly to underlying environmental factors, whereas patterns in neutral alleles respond to varying patterns of demographic and evolutionary (mutation and genetic drift) factors that are only indirectly under environmental influence. Thus, the results of this earlier comparison cannot be extended to neutral genetic data.

The main objective of this paper is to compare the performance of spatial Bayesian clustering methods with that of edge detection methods for identifying genetic boundaries and to provide recommendations for their use. Our comparison includes three published spatial Bayesian clustering approaches and two direct edge detection methods (Table 1). We first compared the methods using data generated by a spatially-explicit simulation model with the objective to evaluate the influence of four factors: (i) time after divergence, (ii) strength of isolation by distance, (iii) level of genetic diversity, and (iv) amount of gene flow across barriers. To estimate the performance of the methods under more typical, “real-world” conditions, we then applied them to two published empirical datasets.

## 2. Materials and Methods

### 2.1. Description of the Methods

Bayesian clustering approaches simultaneously aim to identify the number of clusters and to assign probabilistically either individuals (versions without admixture models) or a fraction of their genome (with admixture models) to identified clusters such that Hardy-Weinberg and linkage disequilibria are minimized. Numerous models and software programs have been developed to achieve these objectives; although their goals are similar (*i.e.*, describing the genetic structure in each subpopulation using a joint probability distribution over the observed loci), the explicit model assumptions, fine details, and computational strategies vary among approaches and may lead to performance differences. In comparison, Monmonier’s algorithm and wombling can be classified as “direct methods” of edge or boundary detection, since they do not look for clusters of individuals but instead focus on boundaries between dissimilar samples. A more detailed technical introduction to these approaches can be found elsewhere [2,16,18,27]. In addition, recent reviews of Guillot *et al.* [8] and Francois and Durand [33] discussed major differences between all of them. For convenience, we will refer to each method by the name of the software that implements it.

The first Bayesian clustering algorithm we consider is BAPS5 [18], which implements both non-spatial and spatial Bayesian clustering algorithms developed by Corander *et al.* [11,18,34,35]. The non-spatial model in BAPS5 is similar to that used by the program STRUCTURE, as implemented by Pritchard *et al.* [9], but in contrast to STRUCTURE, the non spatial model in BAPS5 directly estimates the number of populations [18]. The program has undergone several model and algorithm improvements, in particular a strategy based on a non-reversible Markov chain Monte Carlo algorithm (Table 2). As an extension, the spatial Bayesian clustering algorithm available in BAPS5 uses a Delaunay graph to specify hypothesized connections between individuals or sampling sites based on their locations. The model therefore involves three quantities: a graph specifying the set of neighbors of each individual, a parameter that prescribes the weight of spatial information in the inference scheme, and the number of clusters. The latter two parameters can be inferred within a formal statistical method in BAPS5. The program aims to develop a computationally efficient model requiring minimal user expertise, hence the smaller effective number of parameters (Table 3).

TESS [17,36] uses a hidden Markov Random Field model initially introduced by François *et al.* [16] (Table 2). The model assumes that the log-probability that an individual belongs to a particular cluster given the cluster membership of its closest neighbors is equal to the number of neighbors belonging to this cluster. The probability that two neighboring individuals belong to the same spatial cluster is controlled by a parameter known as the interaction parameter. Any non-zero value introduces spatial dependence, with the default value set to 0.6. In practice, the TESS algorithm involves three quantities: a graph specifying the set of neighbors of each individual, the interaction parameter and the maximum number of clusters. Models with and without admixture are available [36]. When the interaction parameter is set to 0 and the no admixture model is specified, TESS is equivalent to STRUCTURE without admixture and with uncorrelated allele frequencies. The maximum number of clusters that best fit the data is chosen by the user from the statistical Deviance Information Criterion (DIC), while in older versions (prior to version 1.2) this choice was made based on likelihood values. TESS requires testing a range of different values of *Kmax* (the assumed maximum number of clusters) and fixing 6 parameters to estimate the 3 parameters of interest described above (Table 3). This method has been severely criticized by Guillot [37,38] and is the topic of ongoing debate.

GENELAND [15,39–40] implements a method developed by Guillot *et al.* [14]; the program quantifies the amount of spatial dependence in a data set, estimates the number of populations, assigns individuals to their population of origin, and detects individual migrants between populations, while taking into account uncertainty on the location of sampled individuals. The spatial domain of the sample is partitioned into a union of a random number of polygons by Voronoi tessellation that is randomly assigned to one of *K* possible spatial clusters. *K* is considered unknown, with maximal value (*Kmax*) input by users, and estimated by the algorithm. When polygons are assigned to different spatial clusters, the joint probability that any two polygons belong to the same spatial cluster decreases with geographical distance between them [14]. Genetic data can be modeled using either correlated or uncorrelated allele frequencies [8]. Estimates of *K* (the number of spatial clusters) and individual assignment probabilities are obtained using a Metropolis-Hastings algorithm, an iterative procedure which starts from arbitrary values for all unknown parameters and modifies them so that after many iterations they approximate true values.

Monmonier’s algorithm [20] is based on a neighbor graph (such as a Delaunay triangulation) between sampled populations or individuals, and calculates the genetic distances associated with each edge of the graph. The algorithm builds growing barriers from the edge with the largest genetic distance, and extends it to the adjacent edges associated with the next largest genetic distance (see [20,42] for a more extensive description). This algorithm is implemented in the programs Barrier [24], the R package Adegenet [43], and Alleles In Space (AIS, [25]), which we used in this analysis.

Wombling [21] uses surfaces derived from variables of interest (e.g., allele frequency surfaces), and computes gradients for each allele across these surfaces. The gradient norms are then averaged over alleles to form a new surface called the systemic function. Regions given high values by the systemic function can then be identified as zones of rapid change, *i.e.*, boundaries. Various methods have been proposed to estimate the systemic map (e.g., [22]). A significance test (binomial test) on the systemic function has been introduced in the R package WOMBSOFT [27]. WOMBSOFT uses a non-parametric smoothing method originally published by Cercueil *et al.* [26] to interpolate allele frequencies across space. The output includes two maps, showing (1) absolute variations of the systemic function (averaged over loci) in the study area, and (2) areas of sharp allele variation, *i.e.*, boundaries estimated from the significance of the systemic function. The method requires four parameters. The first is the resolution of the grid covering the study area. The second is a user-defined bandwidth, which is typically based on the mean dispersal distance of the species, if known. These two parameters are used to estimate the systemic function in each pixel. The third parameter is a percentile for the systemic function (*p*), and for each allele the method uses the percentile to select candidate boundary elements (*i.e.*, points on the grid where the systemic function is higher than *p*). The last parameter is the significance level, and for each point on the grid the method tests whether the number of candidate boundary elements follows a binomial distribution [B(*n,p*)], where *n* is the total number of alleles in the data set. It is then possible to identify boundary elements that are significant at the chosen significance level.

In this study, we compared the performance of spatial Bayesian clustering algorithms with that of edge detection methods, including (i) the spatial model of BAPS5, (ii) the non-admixture model of TESS, (iii) the admixture model of TESS 2.1, (iv) GENELAND, (v) Alleles in Space, and (vi) WOMBSOFT (Table 1). Next, we describe the simulated and empirical datasets we used to compare the methods.

### 2.2. Simulation Data

Standardized simulated data sets were generated using a time-forward Monte-Carlo procedure that encapsulated and generalized core processes and parameters of evolving spatially-structured populations: (1) organisms inhabit a landscape, (2) each organism is born at a landscape location, (3) distances between birth and breeding sites are a function of dispersal ability, (4) progeny genomes are inherited from parents, and (5) alleles inherited from parents can mutate. Given our interest in simulating barriers to gene flow and exploring emergent patterns associated with genetic discontinuities, our simulations were implemented in a two-step process: (1) simulation of an equilibrium global spatial evolutionary process without barriers, followed by (2) imposition of barriers to organismal movement.

#### 2.2.1. Simulation of Equilibrium Global Spatial Evolutionary Processes

Our simulation procedure was similar to the lattice model implemented in Slatkin and Barton [44,45]. For a given simulated geographical landscape, we specified a 100 by 100 matrix of 10,000 uniformly-ordered individuals. For simplicity, a single diploid, sexually-reproducing hermaphroditic individual capable of acting as either a sperm donor or egg donor inhabits each matrix cell. In each generation, new matrix cells [*x*,*y*] were filled by simulating reproduction between two parents occupying cells from the prior generation. The locations of parents were chosen by randomly selecting a pair of landscape coordinates using a bivariate normal distribution with parameter σ centered on cell [*x*,*y*]. Consequently, the mean axial distance between individual birth and breeding sites (*δ*) is *δ*= *σ√(π/2)* with a standard deviation of *v* = *σ*√(2 – π/2) [46]. Note that larger values of *δ* will permit drawing of parents from a larger spatial extent, and will consequently also reduce the overall degree of spatial genetic structure and isolation-by-distance within the system. In contrast, smaller values of *δ* will restrict the potential set of parents to those closer to cell [x,y], which in turn increases the overall spatial genetic structure and isolation by distance within the simulated landscape. After the selection of parental individuals, a randomly-selected gamete from each parent was used to produce a zygote that inhabits cell [x,y]. We further allowed for mutation of alleles in selected gametes by specifying a uniform pseudorandom number between zero and one. If the pseudorandom number was less than the specified mutation rate (*μ*), then the allele was mutated to a new, previously unobserved allelic state. In our simulations, we initialized all individuals with identical homozygous genotypes (*i.e.*, *H*_0_ = 0, *F*_0_ = 1). However, over the course of many generations, quasi-equilibrium spatial patterns of genetic structure and diversity emerged that reflected specified population sizes and mutation rates. At quasi-equilibrium, spatial distributions of alleles stochastically change over generations, but emergent properties of the system such as number of alleles (*Na*) and heterozygosity (*H*) remain relatively constant. Basic population genetic theory provides a basis for determining expected numbers of alleles and heterozygosity of a population at equilibrium based on population size and mutation rate [47]. In all of our simulation runs, data generated were inspected to ensure that the evolving populations yielded patterns of diversity expected based on specified mutation rates (see below).

#### 2.2.2. Imposing Barriers to Gene Flow

**Figure 1.**
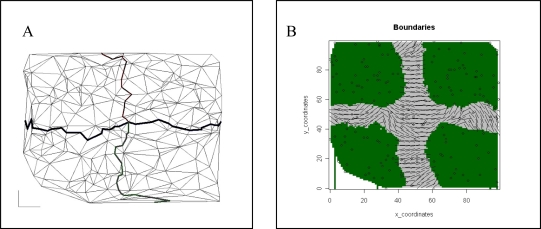

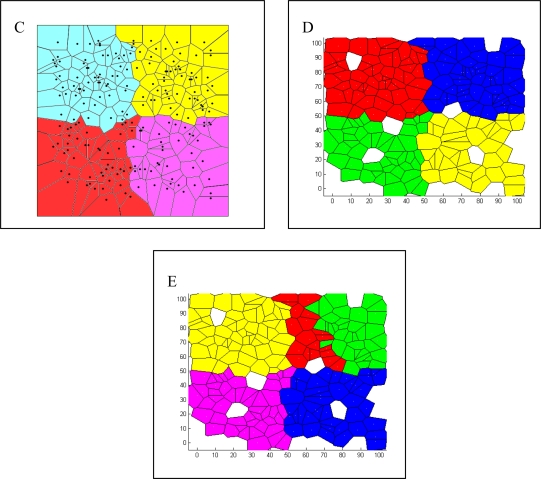
Results obtained with simulated datasets at generation 2,500. Genetic boundaries were simulated using *μ* = 0.000025 and *δ* = 11 for panels A, B, C, and D, and *μ* = 0.000025 and *δ* = 1 for panel e. (**A**) Monmonier's algorithm. Thin lines represent Delaunay triangulation of sampling points. Boundaries are presented as black lines of different width. Wider boundaries are the first detected; (**B**) WOMBSOFT. Circles represent sampling points. Areas in green represent homogenous zones, while boundaries are shown in light grey; (**C**) TESS. Dots represent sampling points. Lines separate Voronoi tessellation polygons. Different colors represent individual spatial cluster membership; (**D**) GENELAND. Dots represent sampling points. Different colors represent individual spatial cluster membership; (**E**) BAPS5. Lines separate Voronoi tessellation polygons. Different colors represent individual spatial cluster membership. BAPS5 overestimated the number of populations mostly for datasets with small dispersal parameters.

Once simulations achieved quasi-equilibrium states, we imposed barriers to gene flow such that the original 100 by 100 landscape was divided into four separate 50 by 50 landscape subsections (see Figure 1). To test the effect of barrier strength, we first assumed that barriers prevented all movement among different landscape subsections. In order to simulate a more realistic scenario, an additional set of simulations was created in which the amount of movement between different landscape subsections is defined by a parameter (*b*) that reflects the proportion of attempted barrier crossings that are allowed in each generation. Note that when *b* = 0, no barrier crossings are permitted and gene flow among landscape subsections is nonexistent.

#### 2.2.3. Simulation Parameters

We examined five simulation parameter combinations that allowed us to evaluate the effects of average dispersal distances (*δ*), mutation rates (μ) and amount of gene flow across barriers (*b*) on the ability of the methods to correctly detect boundaries. Simulation parameters with impermeable barriers (*b* = 0) were as follows: (1) *δ* = 1, *μ* = 10^−4^; (2) δ = 11, *μ* = 10^−4^; (3) *δ* = 1, *μ* = 2.5 × 10^−5^; (4) *δ* = 11, *μ* = 2.5 × 10^−5^. An additional set of simulations was run with 3% barrier permeability (*b* = 0.03), *δ* = 11, and *μ* = 10^−4^. Twenty-five independent simulation replicates were generated for each parameter combination, and 20 unlinked codominant loci were tracked over the course of each simulation replicate. In the case of a population of 10,000 individuals and *μ* = 10^−4^, population genetic theory predicts expected values of *Na* and *H* to be 34.59 and 0.8, respectively, at equilibrium [47]. In the *μ* = 2.5 × 10^−5^ case, *Na* and *H* are expected to be 10.48 and 0.5, respectively. In the initial phase (establishment of quasi-equilibrium patterns of spatial genetic structure), simulations were initially allowed to run for 20,000 and 80,000 generations for mutation rates of *μ* = 10^−4^ and *μ* = 2.5 × 10^−5^, respectively. Simulations were run for more generations in the *μ* = 2.5 × 10^−5^ case because, at low mutation rates, populations will take longer to reach an equilibrium state [47]. In the second phase, genetic data were exported at generations 0 (before barriers were imposed), and at 100, 500, 1000, 3000 and 5000 generations after the barriers were imposed to evaluate the temporal influence of the barrier on genetic structure. Intuitively, as time progresses, the effects of barriers will become more pronounced and result in greater differentiation of landscape regions. At each time point, 200 randomly selected individuals were chosen from the landscape for each data set, and the same randomly selected set of 200 coordinates was used for data sets exported at all six time points (with different sets of coordinates randomly selected for each simulation replicate).

As descriptors of pre- and post-barrier simulation states, we used Arlequin 3.11 [48] to calculate average *F*_ST_ values among landscape subregions at each of the six time points described above for each parameter combination. Note that due to the spatially explicit nature of our simulations, *F*_ST_ values should not be interpreted literally due to the fact that individual landscape subsections are themselves spatially structured, and therefore do not comprise true panmictic populations. Likewise, for the purposes of describing the pre-barrier degree of spatial genetic structure for each parameter set, we followed the general recommendation of Rousset [49] and calculated the average slope of the least-squares regression line derived from the regression of pairwise genetic distances on the log of pairwise geographical distances between each of the 200 individuals sampled from each data set. We expected to see higher regression slopes when the underlying pattern of spatial genetic structure is stronger (*δ* = 1) relative to weaker (*δ* = 11). Mean number of alleles, mean gene diversity and mean isolation by distance (IBD) slope observed in analyzed data sets of 200 individuals for each parameter combination are reported in Table 4. These parameters were not reported for datasets with permeable barriers, since the simulation parameters that influence genetic structure at generation 0 are the same as for the simulations with *δ* = 11, *μ* = 10^−4^ and *b* = 0.

#### 2.2.4. Boundary Detection and Performance Evaluation of Each Method

We applied the five methods to the combination of 5 (parameter combinations) × 6 (generation times) × 25 (replicate simulations per parameter combination) = 750 datasets. The values of the parameters used for the statistical analysis are described in Table 3. Except for generation time 0, where there was no boundary (correct performance = no boundary detection), we expected to have two main boundaries dividing the individuals into four separate spatial groups (Figure 1). The following main steps were followed when evaluating the Bayesian clustering methods:

1. For each data set, we performed 10 independent runs for values of *Kmax* ranging from 1 to 6 (Table 3). In the case of TESS, values of *K* corresponding to the highest average likelihood score across runs were identified. Outputs from TESS analyses using the identified value of *K* were averaged over the 10 runs using the computer program CLUMPP [50]. BAPS5 provides its own internal approach for inferring the most likely number of clusters in a data set, presenting only the result with the highest average likelihood score and the corresponding cluster membership coefficients of each individual across the 10 runs.
2. For Bayesian clustering methods, we considered boundaries to be correctly inferred if the number of spatial clusters estimated was four and if individuals were all correctly assigned to the clusters from their respective landscape region, except for generation time 0. At generation 0, we expected no detectable boundary and for all individuals to be assigned to the same cluster (*K* = 1).
3. For each parameter combination and at each time point, we calculated the percentage (out of the 25 repetitions) of cases where the boundaries (or no boundary at generation 0) had been correctly detected.

For both edge detection methods, we visually checked outputs from each program to determine if boundaries were correctly detected (out of the 25 repetitions performed for each parameter set); Figure 1A and B show results considered to be correct.

### 2.3. Empirical Data

To evaluate the performance of the methods for the empirical datasets, we reanalyzed two previously published datasets and compared our results with those reported in the original papers.

#### 2.3.1. Puma (Puma Concolor)

The first dataset consisted of 540 pumas from the southwestern United States (Latitude: 31° to 42°, Longitude: −114° to −103°) genotyped for 16 microsatellite loci by McRae *et al.* [13]. The number of detected alleles per locus was 3.95, and average expected heterozygosity was 0.62. We reanalyzed the data using BAPS5, TESS, GENELAND, WOMBSOFT and AIS. In TESS, values for parameters of MCMC chain (number of sweeps and burn-in number of sweeps) and prior distribution (maximal number of clusters and interaction parameter) were chosen through a series of evaluation runs with varying values. Values of the chain length and the burn-in period for which convergence was reached, the value for maximal numbers of spatial clusters (from 1 to the number of populations presented in the original paper increased by 2) and the interaction parameter (Table 3) that gave the highest likelihoods were chosen and used for the 10 independent final runs performed with and without the admixture option. For BAPS5, we evaluated the same range of *Kmax* values as for TESS (Table 3). Ten runs of GENELAND model with uncorrelated allele frequencies were applied. *Kmax* values for the runs were chosen based on the results of the original paper and compared to the estimated number of spatial clusters from each run in order to avoid limiting estimation by setting overly small values. For WOMBLING, the grid resolution and the bandwidth size were chosen from the size and the shape of the sampling area. Monmonier’s algorithm was applied on both raw and residual genetic distances to remove the influence of isolation by distance [24]. Up to five boundaries were specified for the analysis.

#### 2.3.2. Rhododendron (Rhododendron Ferrugineum)

The second dataset consisted of leaf samples of R. ferrugineum collected during summer of 2004 across the entire European Alps (latitude: 44°48′ to 48°36′; longitude: 5°20′ to 15°40′). A 12′ latitude × 20′ longitude (*ca.* 23 km × 25 km) grid was adopted and three plants were sampled in each of 127 cells resulting in a total of 380 samples distributed over *ca*. 171,350 km^2^ (see [51] for more details). One hundred and twenty AFLP markers were generated following the protocol of Vos *et al.* [52]. These data were first analyzed with methods adapted for dominant markers (BAPS5, GENELAND, WOMBSOFT, AIS), considering AFLP markers as binary variables [53]. To allow the application of TESS to these dominant data, we recoded them as suggested by Evanno *et al.* [54], coding each individual with band absence as a recessive diploid homozygote (0-0 genotype, each number corresponding to an allele) and each individual with band presence as either dominant homozygote or heterozygote (1–99 genotype, 99 indicating missing data). The resolution of the grid used in WOMBSOFT was the same as the sampling grid (*i.e.*, 23 km × 25 km), with a slightly larger bandwidth of 30 km (Table 3). For all the other parameters (convergence parameters), we used the same criteria as those used for the puma dataset.

## 3. Results

### 3.1. Simulated Data

Over all simulation parameter combinations (including permeable and non-permeable barriers), the mean percentage of cases where the boundaries were correctly identified was highest for GENELAND and lowest for WOMBSOFT (Table 5). The Bayesian clustering methods without admixture outperformed edge detection methods in all cases. The success rate and order in which the methods ranked in their ability to correctly detect boundaries is: GENELAND (69%), TESS (spatial, no admixture; 57%), BAPS5 (38%), Monmonier’s algorithm (27%), TESS (spatial with admixture 25%), WOMBSOFT (21%) (see Table 5). For datasets simulated with non-permeable barriers only, the rankings change in favor of TESS with no admixture (67%).

At generation 0, all spatial variation should occur across gradients and is due to isolation by distance, and no sharp boundaries should be detected. Our simulations showed significant isolation by distance patterns (larger slopes) with *δ* = 1 relative to simulation where dispersal was more widespread (*δ* = 11) (Table 4). However, both edge detection methods and TESS with admixture always detected boundaries (Table 5), despite the fact that no barriers were yet imposed upon the landscape. The other Bayesian clustering methods (TESS without admixture, GENELAND and BAPS5) correctly identified a single cluster (no boundaries at generation 0) only when the overall degree of spatial genetic structure was low (*i.e.*, *δ* = 11) (Table 5).

**Figure 2.**
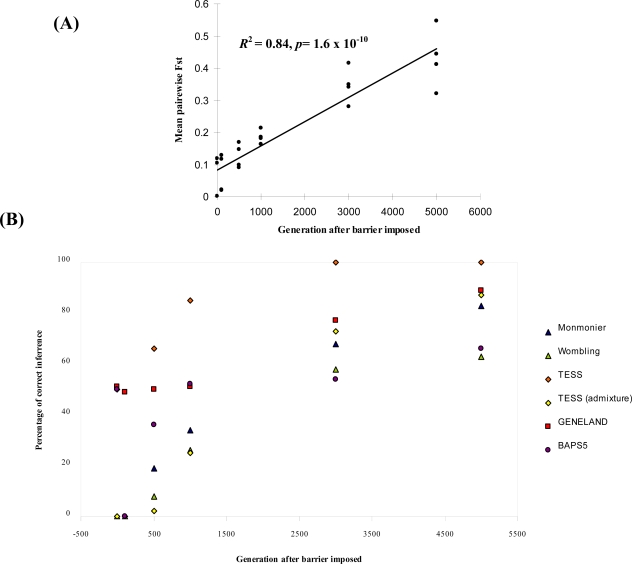

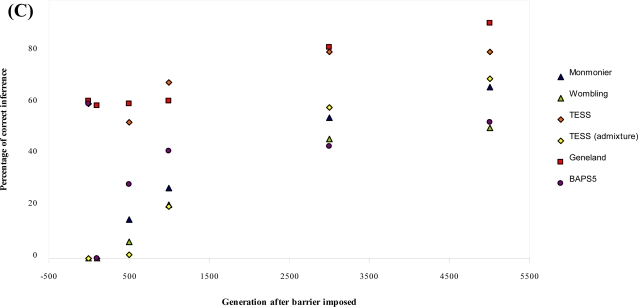
(**A**) Relationship between global F*_ST_* (averaged across repetitions) and generation after imposition of barriers for each of four simulated datasets with impermeable barriers, *b* = 0. (**B**) Percentage correct inference averaged across dispersal and mutation rates in relation to generation time for simulated datasets with impermeable barriers, *b* = 0. (**C**) Percentage correct inference averaged across dispersal and mutation rates in relation to generation time for simulated datasets with permeable boundaries (*b* = 0.03).

As expected, the time elapsed (number of generations) following imposition of barriers was highly correlated with F*_ST_* (Figure 2A). Likewise, the overall success rate of each procedure tended to increase with time following barrier imposition (Table 5). For non-permeable barriers, at generation 100, GENELAND correctly detected boundaries in 49% of datasets, while no boundaries were detected by any other procedure (Figure 2B). However, the percentage of correct boundary detections increased with generation time across methods, becoming highest for TESS without admixture. Above generation 3000, TESS detected boundaries with at least 60 % success, and this increased to 100% at generation 5000 (Table 5, Figure 2B). All of the Bayesian clustering methods had a high success (average of 83.3%) for generations > 1000, while edge detection methods were successful in less than 50% of cases for the same datasets. Differences in performance among the methods depended highly on the parameter set under which the data were simulated (Table 5). At higher dispersal distances (*δ* = 11; lower spatial genetic structure), boundaries were better detected by all methods for generation 500 and later (Table 5). Decreasing the mutation rate decreased the mean percentage of boundary detections except for *δ* = 1 for BAPS5 and WOMBSOFT (Table 5).

The lower ranking of BAPS5 relative to the two other Bayesian clustering methods resulted from poor performance under parameter combinations with *δ* = 1, where BAPS5 never inferred the correct structure but instead always overestimated the number of clusters (Table 5; see Figure 1E for an example).

For the datasets with permeable barriers (*b* = 0.03), only GENELAND was able to correctly detect boundaries in all datasets. This increased its overall performance above TESS when the results for all parameter combinations and dispersal scenarios were compared. TESS with no admixture and BAPS5 correctly detected only absence of boundaries for generation 0 in all 25 replicates (17%), while for all other settings they detected only up to three spatial clusters. Edge detecting methods failed to detect correct boundaries in all datasets with *b* = 0.03.

### 3.2. Empirical Data

Although TESS and BAPS5 identified boundaries between clusters that were largely consistent with the original studies, results still varied between the two methods, with different numbers of clusters identified for both species. Using the admixture option in TESS increased the number of identified clusters. Results of GENELAND were in both cases almost identical to the results of BAPS5. The WOMBSOFT results were difficult to interpret, identifying large heterogeneous areas whereas Monmonier’s algorithm never identified biologically interpretable boundaries, instead detecting individuals that were more genetically differentiated from their neighbors than expected (possible migrants).

#### 3.2.1. Puma

**Figure 3.**
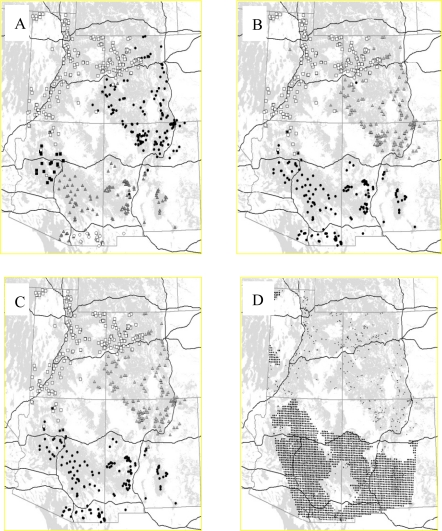
Results for puma dataset. (**A**) Five clusters identified by TESS. (**B**) Three clusters identified by BAPS5. (**C**) Three clusters identified by GENELAND. (**D**) Significant boundary elements (black circles) detected by WOMBSOFT. Small dots indicate sampling sites. Puma habitat is shown in grey.

All Bayesian approaches identified the strong boundary between northern and southern puma populations reported by the original authors (Figure 3). In all the evaluation runs for models with and without admixture, TESS did not detect more than seven spatial clusters, so for the 10 final runs, seven was chosen as maximum number of populations, and the interaction parameter was set to 1 according to the highest likelihood score for this value in the evaluation runs. In all the results of the 10 final runs, TESS with admixture detected four to five spatial clusters, with similar north-south separation. Processing those results with CLUMPP gave a solution with five spatial clusters (Figure 3A). The two northern clusters found by TESS highly corresponded to the BAPS5 solution that found only three clusters (Figure 3B), with TESS detecting some additional differentiation in the south. GENELAND detected two to three spatial clusters in all 10 runs, and the run with highest likelihood had three. The locations and individual assignments (Figure 3C) of those spatial clusters closely resembled to BAPS5 solution, with the exception of some differently assigned individuals in the zone of contact between clusters. WOMBSOFT indicated a heterogeneous area in the south (Figure 3). Monmonier’s algorithm identified locally differentiated individuals or small groups not interpretable as boundaries (results not shown).

#### 3.2.2. Rhododendron

**Figure 4.**
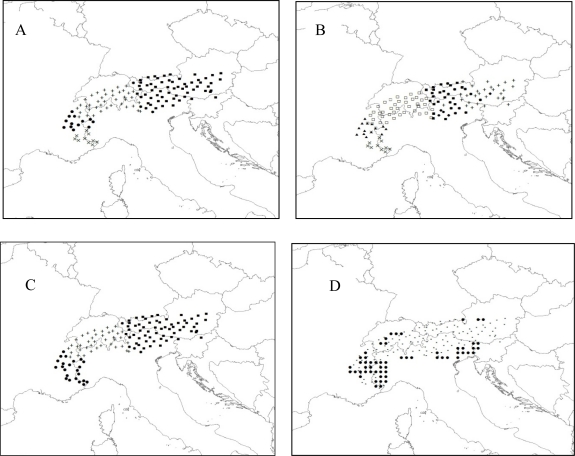
Results for Rhododendron dataset. (**A**) Six clusters identified by TESS; (**B**) Four clusters identified by BAPS5; (**C**) Three clusters identified by GENELAND; (**D**) Significant boundary elements (black circles) detected by WOMBSOFT. Small crosses indicate sampling sites.

TESS without admixture detected six spatial clusters among the *R. ferrugineum* samples (Figure 4A) while BAPS5 identified four (Figure 4B) and GENELAND identified three (Figure 4C). Although TESS is not specifically designed for the analysis of dominant markers, two clusters in the southeast and the large middle cluster (located mostly in Swiss Alps) were congruent with clusters detected by BAPS5, while TESS detected more clusters in the western part of sampling area than did BAPS5. Results of GENELAND matched very well with BAPS5, with two smaller western spatial clusters merged into one with GENELAND. As with the puma data, the WOMBSOFT results were difficult to interpret, indicating a heterogeneous area in the south and boundaries on the edge of the studied area (Figure 4C) [51]. Monmonier’s algorithm detected long barriers separating individuals that were not biological neighbors, but were connected in the neighborhood graph by the algorithm (results not shown).

## 4. Discussion

In this paper, we compared five methods with different underlying models in their efficiency and reliability for detecting genetic boundaries in both ideal (simulated) and realistic cases. Our simulations allowed us to compare the results from each method against barrier locations known with perfect certainty, and to evaluate effects of factors such as time since barrier creation, mutation rate, dispersal distance, and gene flow across the barrier on the ability of each method to correctly infer boundaries. Our empirical analyses allowed a more realistic test of each method’s performance, introducing complexities commonly found in real-world studies, including complicated and unknown population histories, irregular and ad-hoc sampling schemes (pumas), and genetic markers that do not conform to some of the methods’ assumptions (AFLPs). We do not claim to be exhaustive, but to present results from a set of cases carefully chosen to shed light on a rapidly expanding research area.

The most striking result of our simulations was that the spatial Bayesian clustering methods outperformed the direct edge detection methods. While TESS matched or outperformed the other spatial models for datasets with small dispersal distances (*δ* = 1), GENELAND performed best for datasets with *δ* = 11 and permeable barriers, identifying boundaries with almost 100% accuracy. BAPS5 performed well except under parameter combinations with *δ* = 1, where it rarely inferred the correct structure. For the same parameter combination, BAPS5 and GENELAND consistently tended to detect more than four simulated spatial clusters, finding additional subdivisions within clusters. Although visual inspection of these results indicated that in most cases the location of the simulated barrier was correctly detected, they could not be counted as correct using our criteria. Small dispersal distances and a high mutation rate in this scenario likely combined to result in spatial aggregation of similar alleles, giving the illusion of movement barriers when none actually existed [41]. This inference is in agreement with recently published results of Frantz *et al.* [32], who showed that both GENELAND and BAPS5 can overestimate genetic structure in datasets characterized by isolation by distance. They did not include TESS in their study, so their results cannot be extended to it without further evaluation under different isolation by distance scenarios.

Under our simulation parameters TESS with admixture generally performed poorly, an unsurprising result given that the imposition of impermeable barriers meant that there was no ongoing migration between subpopulations, and thus admixture was not to be expected. This poor performance can also be explained by the fact that using the admixture option in TESS increases the number of parameters to estimate, reducing the reliability of parameter estimations in the absence of admixture. This has been recently tested by Francois and Durand [33] in a review paper related to spatial Bayesian clustering method.

The success of GENELAND and failure of other methods for simulated datasets with permeable barriers used in this study suggests that GENELAND is better suited for migration scenarios that may be common in empirical datasets. Movement across barriers, in combination with large dispersal distances, allows for strong gene flow across entire landscapes, and as a consequence weak spatial structure that is difficult to detect. Poor results by TESS for permeable barriers can further be explained by its lack of a correlated allele frequencies model; this model better matched our simulations because of the instantaneous population fission scenario we used.

Another general result from our simulations was the presence of time lags between barrier imposition and their reliable detection by all methods (Table 5, Figure 2B). In our case the time lag is substantial. The results of a recent study on simulated datasets [55] suggested that barriers can in some cases be detected after as few as 15 generations. However, those results are not fully comparable with ours due to different simulation scenarios, a different approach to the testing the barrier effect, and different objectives. In our case, we started by simulating an underlying isolation by distance pattern, and then imposed barriers onto the landscape, whereas Landguth *et al.* [55] generated a random distribution of genotypes across the landscape, then simultaneously imposed both barriers and restricted dispersal. That means that the isolation by distance process was not independent of the barrier effect, and that it therefore could have also contributed to the statistical signal that they detected. Furthermore, their barrier detection approach was based on use of partial Mantel tests, which required specification of the location of an *a priori* hypothesized barrier. In our study we only included methods that can detect barriers without an *a priori* notion of where they occur. In natural populations, the length of time lags can also be related to sampling scheme, effective population size and substructure [33,56,57].

Our empirical analyses also give more support to the clustering methods than to the direct edge detection methods. Monmonier’s algorithm, using both raw and residual genetic distances for both datasets, only detected boundaries around particular individuals that were genetically highly distinct from others in their immediate vicinity. Wombsoft detected, for both empirical datasets, wide heterogeneous areas difficult to interpret as boundaries. Although the exact numbers of spatial clusters detected by the Bayesian spatial clustering methods differed for empirical datasets, all of them gave solutions that were consistent with those reported in the original papers. For both datasets, results from GENELAND and BAPS5 matched very closely. For the puma dataset, both methods detected three spatial clusters and the same boundaries between northern and southern clusters as reported by McRae *et al.* [13]. Although TESS detected more clusters, the location of the strong north-south boundary is unchanged. For the rhododendron dataset, the methods detected two strong genetic discontinuities isolating three putative populations [51]. Two of these populations—one in the eastern Alps and one in the central Alps—were very homogeneous. The third, located in the southwestern Alps, was less homogeneous [51], perhaps explaining the additional boundary identified by BAPS5 and TESS. The additional boundary identified by TESS in the north was detected by no other methods and thus difficult to trust. Additional local analyses would be required to confirm the observed structure.

WOMBSOFT performed particularly poorly with our simulated datasets, and both edge detection algorithms performed poorly with the empirical datasets. While wombling proved its potential in ecology [57] and in spatial genetics [58] as did Monmonier’s algorithm with population-based genetic datasets [24,28,29], we cannot recommend their use with individual-based genetic data given our results. The decreased performance of the edge detection methods with individual-based genetic data is likely due in part to differences in the sources of variation between the data types. Sampling in ecology is affected by individual- and population-level variation, whereas genetic data contain additional variation caused by sampling of loci within individuals. This within-individual variation is often very high in relation to other levels, introducing additional noise into individual-based analyses. The variance in individual genotypes can particularly explain the poor performance of Monmonier’s algorithm, which is in essence a local edge detector which is especially sensitive to local maxima when identifying boundaries [28].

Neutral genetic markers do not respond directly to underlying environmental factors. Boundaries detected in data based on species occurrence likely reflect real factors to which the species are responding. For example, an individual sample of plant community data surrounded by an inferred boundary likely indicates a localized area where environmental conditions (e.g., soil type) have resulted in a distinct community of species [19]. Conversely, a similar boundary detected in individual genetic data may only denote an individual that is distinct from its neighbors, either due to random sampling variance in its genotype or a long distance dispersal event. Because wombling operates on surfaces which locally integrate genetic data across many individuals, this technique may be less sensitive to individual variation than Monmonier’s algorithm. Nonetheless, wombling has performance limitations as our results show.

## 5. Conclusions and Recommendations

Based on the percentage of correct inference for simulated data (Table 5), and comparison with previously published results for empirical datasets, we recommend the use of Bayesian clustering methods over local edge detection methods for individual-based genetic analyses. All algorithms are especially powerful in detecting spatial genetic structure, although spatial autocorrelation and isolation by distance may inflate the true number of clusters when dispersal distances are limited. Based on the analyses of our simulated datasets that include gene flow across barriers, GENELAND seems the most appropriate overall approach. Our results lend further support to the idea that a combination of different analysis techniques can provide complimentary information and elucidate important patterns in individual-based population genetic data. While the performance of the local edge detection methods was poor (particularly for the empirical datasets), there is still a need for the development of analytical approaches that can provide additional information about the location and shape of genetic boundaries [59]. Such approaches, not based on specific genetic assumptions, will be especially useful for mating systems that deviate from Hardy Weinberg equilibrium or for polyploid organisms.

In natural populations, substructuring of individuals can be caused by influences of isolation by distance, gradients of landscape resistance, and true barriers [4]. The methods we compared were not able to separate these effects, so we advise additional tests to avoid misinterpretation of results. When investigating genetic boundaries in a dataset, we recommend first determining if isolation by distance patterns exists. If isolation by distance is not present, then any of the evaluated spatial clustering methods can be used. If isolation by distance is significant, our results from simulated data suggest that TESS (with CLUMPP) will be more powerful, but this must be balanced against the potential advantages of GENELAND discussed above. Lastly, when working with TESS, our analyses suggest that the use of the admixture model reduces the program’s ability to detect barriers when there is no admixture. We therefore advise researchers to carefully consider their hypotheses regarding ongoing migration between subpopulations before deciding whether to include admixture in their analysis model.

## Figures and Tables

**Table 1.** Main characteristics of software packages compared in this study.

	**BAPS5**	**TESS**	**GENELAND**	**WOMBSOFT**	**AIS (Monmonier)**
Model	Spatial Bayesian clustering	Spatial Bayesian clustering	Spatial Bayesian clustering	Non parametric	Non parametric
	Analytical and Stochastic methods	Markov chain Monte Carlo	Markov chain Monte Carlo		

Spatial	Colored Voronoi tessellation based on discrete sampling site	Hidden Markov random field	Free colored Voronoi tessellation based on continuous Poisson point process	Geographic coordinates are included in the local weighted regression	Delaunay triangulation (Table 5)

Clustering criteria	*HWE and **LE between loci	*HWE and **LE between loci	*HWE and **LE between loci	None	None

Local edge detection criteria	None	None	None	Average rate of change based on individuals located within a kernel of a given bandwidth size	High rate of change between paired individuals based on Delaunay link

Data	Co-dominant and Dominant	Codominant	Co-dominant and Dominant	Co-dominant, Dominant and categorical data	Co-dominant, Dominant, and Sequence Data

Platforms	Windows, Unix/Linux Mac OS X	Windows, Unix/Linux	R package Windows, Unix/Linux Mac OS X	R package Windows, Unix/Linux Mac OS X	Windows

Reference	[35]	[16,17]	[15]	[27]	[25]

URL	http://web.abo.fi/fak/mnf//mate/jc/software/baps.html	http://www-timc.imag.fr/Olivier.Francois/tess.html	http://www2.imm.dtu.dk/~gigu/Geneland/	http://cran.r-project.org/web/packages/wombsoft/index.html	http://www.marksgeneticsoftware.net/AISInfo.htm

* HWE: Hardy Weinberg equilibrium

** LE: linkage equilibrium

**Table 2.** Glossary of technical terms used.

Hidden Markov Random Field	A hidden Markov random field model is a special case of Hidden Markov Models (HMM). A HMM is a statistical model in which the system being modeled is assumed to be a Markov process with unknown parameters, and the challenge is to determine the hidden parameters from the observable parameters.

Markov chain Monte Carlo	Markov chain Monte Carlo methods are a class of algorithms for sampling from probability distributions based on constructing a Markov chain that has the desired distribution as its equilibrium distribution.

Neighborhood graphs	Neighborhood graphs capture proximity between points by connecting nearby points with a graph edge. Many possible ways to determine nearby points lead to a variety of neighborhood graph types such as Voronoi tesselation and Delaunay triangulation.

Voronoi tesselation	Given a set of *N* points in a plane, Voronoi tessellation divides the domain in a set of polygonal regions, the boundaries of which are the perpendicular bisectors of the lines joining the points.

Delaunay triangulation	The Delaunay triangulation graph connects the adjacent geographical positions of the samples on a map, resulting in a network that connects all the samples. None of the points is inside the circumcircle of any triangle.

**Table 3.** Input parameters used for Bayesian clustering methods, WOMBSOFT and Monmonier’s algorithm (AIS) in our application.

	**Input parameter**	**Simulations**	**Puma**	**Rhododendron**
BAPS5	*K	1–6	1–8	1–10
	Number of replications	10	10	10

TESS	*K	1–6	1–7	1–7
	**Psi:	0–0.6	0.6–1	0.6–1
	Number of Sweeps	10,000	100,000	100,000
	Number of burnin period	2000	10,000	10,000
	Number of runs	10	10	10
	Admixture parameter	†Yes and no	Yes and no	Yes and no

GENELAND	*K	1–6	1–7	1–7
	Number of iterations	50,000	100,000	100,000
	Thinning	10	10	10
	Number of replications	10	10	10
	Allele frequencies	Correlated	Correlated	Correlated

WOMBSOFT	Resolution of the grid	100 × 100	100 × 100	34 × 16
	Bandwidth	7	70 km	30 km
	Binomial threshold	0.3	0.3	0.3
	Statistical significance of the binomial test	0.05	0.01	0.05

Monmonier’s algorithm	Genetic distances were specified	Residual	Raw and residuals	Raw and residuals
	Number of barriers to be identified.	4	1–7	1–7

* *K*: maximal number of clusters.

** Psi: the interaction parameter of TESS can be interpreted as the intensity with which two neighbors belong to the same clusters. The higher the value of psi is the more likely the population may consists of a unique cluster with a high level of genetic continuity.

† Admixture model was used although we know that our data have no admixture.

**Table 4.** Mean number of alleles, mean gene diversity and mean isolation by distance (IBD) slope observed in analyzed data sets of 200 individuals for each parameter combination. The mean IBD slope was calculated using replicate data sets from the pre-barrier stage (generation 0). Standard deviations for all values are indicated in brackets. Mutation rates are given by μ and average dispersal distances by δ. In parentheses, the percentage of individual tests for each parameter combination that gave significant slopes at the α = 0.05 level is shown.

**Parameter combination**	Mean Number of alleles*	Mean gene diversity (H)*	**Mean IBD Slope†**
*μ* = 0.0001 − *δ* = 1	24.9 [0.59]	0.86 [0.0088]	0.265 [0.02] (100%)
*μ* = 0.000025 − *δ* = 1	9.5 [0.68]	0.62 [0.0073]	0.202 [0.03] (100%)
*μ* = 0.0001 − *δ* = 11	17.8 [1.28]	0.79 [0.0182]	0.004 [0.003] (12%)
*μ* = 0.000025 - *δ* = 11	6.32 [0.36]	0.49 [0.0098]	0.002 [0.002] (24%)

* Calculated over generation time and over repetitions.

† Calculated only for generation time 0 over repetitions.

**Table 5.** Percent correct inferences observed in simulated data for each parameter combination (25 replicates in each case) and for each generation time. At *t* = 0, we consider the inference to be correct if no boundary has been detected. For all the other generation times, inferences are correct if the two main boundaries to gene flow have been detected (*i.e.*, *K* = 4). *μ* reflects mutation rate, *δ* average dispersal distances, *b* boundary permeability (*i.e.*, gene flow across the boundary). The mean number of clusters (averaged over replicates) estimated by Bayesian clustering methods are reported in brackets.

**Parameter combination**	**Generation after barrier imposed**	**Monmonier**	**WOMBSOFT**	**TESS**	**TESS admixture**	**GENELAND**	**BAPS5**
*μ* = 0.0001	0	0	0	0 [5.4]	0 [5.2]	0 [2.0]	0 [6.0]
*δ* = 1	100	0	0	0 [5.2]	0 [4.8]	0 [5.2]	0 [6.0]
*b* = 0	500	36	0	72 [4.3]	4 [4.6]	0 [5.2]	0 [6.0]
	1000	68	8	84 [4.2]	52 [4.1]	4 [5.1]	0 [6.0]
	3000	96	20	100 [4.0]	100 [4.0]	60 [4.5]	0 [5.9]
	5000	100	24	100 [4.0]	92 [4.0]	88 [4.1]	0 [5.9]
*μ* = 0.000025	0	0	0	0 [5.4]	0 [4.9]	4 [5.0]	0 [6.0]
*δ* = 1	100	0	0	0 [5.5]	0 [4.6]	0 [5.0]	0 [6.0]
*b* = 0	500	4	0	36 [4.7]	0 [4.6]	0 [5.2]	0 [5.9]
	1000	8	0	68 [4.3]	12 [4.3]	0 [5.6]	12 [5.6]
	3000	40	28	100 [4.0]	60 [4.1]	48 [4.7]	16 [5.2]
	5000	68	40	100 [4.0]	80 [4.0]	68 [4.4]	64 [4.5]
*μ* = 0.0001	0	0	0	100 [1.0]	0 [3.5]	100 [1.0]	100 [1.0]
*δ* = 11	100	0	0	0 [2.6]	0 [3.9]	100 [4.0]	0 [1.0]
*b* = 0	500	36	32	100 [4.0]	4 [4.0]	100 [4.0]	100 [4.0]
	1000	60	72	100 [4.0]	32 [4.0]	100 [4.0]	100 [4.0]
	3000	96	92	100 [4.0]	92 [4.0]	100 [4.0]	100 [4.0]
	5000	100	88	100 [4.0]	96 [4.0]	100 [4.0]	100 [4.0]
*μ* = 0.000025	0	0	0	100 [1.0]	0 [3.6]	100 [1.0]	100 [1.0]
*δ* = 11	100	0	0	0 [1.4]	0 [3.8]	96 [4.0]	0 [1.0]
*b* = 0	500	0	0	56 [4.0]	0 [4.0]	100 [4.0]	44 [4.0]
	1000	0	24	88 [4.0]	4 [4.0]	100 [4.0]	96 [4.0]
	3000	40	92	100 [4.0]	40 [4.1]	100 [4.0]	100 [4.0]
	5000	64	100	100 [4.0]	80 [4.0]	100 [4.0]	100 [4.0]
**Mean % (*b* = 0)**		**27.20**	**20.67**	**56.80**	**24.93**	**68.93**	**37.73**
*μ*= 0.0001	0	0	0	100 [1.0]	0 [2.4]	100 [1.0]	100 [1.0]
*δ* = 11	100	0	0	0 [1.0]	0 [2.3]	100 [4.0]	0 [1.0]
*b* = 0.03	500	0	0	0 [1.9]	0 [2.3]	100 [4.0]	0 [1.0]
	1000	0	0	0 [1.9]	0 [2.4]	100 [4.0]	0 [1.0]
	3000	0	0	0 [2.0]	0 [2.6]	100 [4.0]	0 [1.0]
	5000	0	0	0 [2.0]	0 [2.9]	100 [4.0]	0 [1.0]
**Overall Mean %**		**34.00**	**25.83**	**66.83**	**31.16**	**61.17**	**43.00**
